# Development of a blood-based extracellular vesicle classifier for detection of early-stage pancreatic ductal adenocarcinoma

**DOI:** 10.1038/s43856-023-00351-4

**Published:** 2023-10-19

**Authors:** Juan Pablo Hinestrosa, Rosalie C. Sears, Harmeet Dhani, Jean M. Lewis, Gregor Schroeder, Heath I. Balcer, Dove Keith, Brett C. Sheppard, Razelle Kurzrock, Paul R. Billings

**Affiliations:** 1 Biological Dynamics Inc, San Diego, CA USA; 2 Department of Molecular and Medical Genetics, Brenden-Colson Center for Pancreatic Cancer, Knight Cancer Institute, Oregon Health and Sciences University, Portland, OR USA; 3 Brenden-Colson Center for Pancreatic Cancer, Knight Cancer Institute, Oregon Health and Sciences University, Portland, OR USA; 4 Medical College of Wisconsin, Milwaukee, WI USA; 5 Worldwide Innovative Network for Personalized Cancer Medicine, Chevilly-Larue, France

**Keywords:** Pancreatic cancer, Cancer screening

## Abstract

**Background:**

Pancreatic ductal adenocarcinoma (PDAC) has an overall 5-year survival rate of just 12.5% and thus is among the leading causes of cancer deaths. When detected at early stages, PDAC survival rates improve substantially. Testing high-risk patients can increase early-stage cancer detection; however, currently available liquid biopsy approaches lack high sensitivity and may not be easily accessible.

**Methods:**

Extracellular vesicles (EVs) were isolated from blood plasma that was collected from a training set of 650 patients (105 PDAC stages I and II, 545 controls). EV proteins were analyzed using a machine learning approach to determine which were the most informative to develop a classifier for early-stage PDAC. The classifier was tested on a validation cohort of 113 patients (30 PDAC stages I and II, 83 controls).

**Results:**

The training set demonstrates an AUC of 0.971 (95% CI = 0.953–0.986) with 93.3% sensitivity (95% CI: 86.9–96.7) at 91.0% specificity (95% CI: 88.3–93.1). The trained classifier is validated using an independent cohort (30 stage I and II cases, 83 controls) and achieves a sensitivity of 90.0% and a specificity of 92.8%.

**Conclusions:**

Liquid biopsy using EVs may provide unique or complementary information that improves early PDAC and other cancer detection. EV protein determinations herein demonstrate that the AC Electrokinetics (ACE) method of EV enrichment provides early-stage detection of cancer distinct from normal or pancreatitis controls.

## Plain language summary

Pancreatic cancer is one of the deadliest cancers and it is often detected when it is too late, limiting treatment options and reducing survival rates. Identifying blood-based markers of pancreatic cancer may help us to diagnose it earlier, when it is more treatable. Tiny particles circulating in the blood stream, called extracellular vesicles (EVs), contain useful information about tumors, which can be collected with our innovative technology. In this study, we analyzed markers present within EVs and developed a computational tool using this information to identify people with early-stage pancreatic cancer. With further testing in real-world settings, this approach may prove useful for surveillance and early detection of this deadly disease.

## Abstract (web summary)

Hinestrosa et al. developed a machine learning classifier for the detection of early-stage pancreatic cancer using a blood-based extracellular vesicle protein assay. The algorithm successfully classifies cancer cases versus healthy and pancreatitis controls with high sensitivity and specificity.

## Introduction

Pancreatic cancer was the third leading cause of cancer-related death in the United States in 2022 with 49,830 deaths; an estimated 62,210 additional Americans were diagnosed during this time, and 75% of these patients will not survive beyond 12 months of the initial diagnosis^1^. By 2030, pancreatic cancer is expected to surpass colorectal cancer in becoming the second leading cause of death^2,3^. Pancreatic Ductal Adenocarcinoma (PDAC) is the most prevalent type, accounting for greater than 90% of all pancreatic cancer malignancies^4,5^.

The United States Preventive Services Task Force (USPTF) does not endorse screening for pancreatic cancer for the average-risk population but does recommend surveillance for patients at high risk due to inherited genetic syndromes (e.g., Peutz–Jeghers syndrome, hereditary pancreatitis) or with a familial history of pancreatic cancer. Other known PDAC risk factors that have not been supported for surveillance by the USPTF include new-onset diabetes (NOD) in adults over 50 years old, pathogenic germline mutations (ATM, BRCA1/2, CDKN2A, MLH1, MSH2, MSH6, PALB2, STK11, TP53), obesity, age, and pancreatic cysts such as intraductal papillary mucinous neoplasms (IPMNs). For patients with a pathogenic germline mutation, current International Cancer of the Pancreas Screening (CAPS) guidelines indicate that these patients should undergo pancreatic surveillance only if there is also familial history of PDAC^6^. Interestingly, 78–91% of the PDAC patients who carry these mutations do not have the family history and thus are not recommended for PDAC surveillance by most current professional society guidelines; nevertheless, these patients still possess an elevated disease risk^7,8^.

Surveillance of patients with an elevated risk for PDAC, primarily with clinical and imaging modalities, could potentially improve outcomes; however, effective surveillance programs face several challenges, including patient compliance, disparities in healthcare, proximity to high-quality imaging centers, and the potential for malignancy to develop between imaging events^9–12^. Several studies have shown that diagnosis of pancreatic cancer at the local stage is severely limited for healthcare-vulnerable patients^13–15^. Given these impediments, and the high mortality of PDAC, there is a need to develop easily accessible approaches with the ability to identify PDAC at its earliest stages^10^.

Recently, the use of blood-based biomarkers to detect cancer, commonly known as liquid biopsy, has expanded with the advent of new technologies and innovative research^16–19^. The concept of liquid biopsy was developed to address the need for minimally invasive testing, particularly in situations where tumor tissue is not readily available. Liquid biopsy now encompasses a range of methods that involve the isolation and study of tumor-derived molecules including DNA, RNA, and protein. Extracellular Vesicles (EVs), including exosomes, are among the most promising sources of biomarkers providing a full spectrum of informative cellular components - DNA, RNA, metabolites, and proteins^20–22^. EVs have been shown to mediate cell-to-cell communication, and play an important role in regulating tumor malignancy^23,24^. EVs, in particular those from 50 to 200 nm in size, have been shown to promote tumorigenesis and carry functional protein biomarkers representing the tumor proteome making them suitable candidates for their application in liquid biopsies, including early cancer detection^20,24–26^. Thus, EVs represent a prime source for the enrichment and detection of cancer-specific biomarkers for multiple solid tumors, including pancreatic cancer^27,28^.

We previously demonstrated that the Verita™ platform, based on the alternating current electrokinetics (ACE) technology, efficiently isolates EVs, including exosomes, from plasma samples in the range of 50 to 200 nm^29,30^ and has been shown to have applications in multiple areas^31–36^. This approach was used to compare EV-protein concentrations across case and control groups and develop a machine-learning algorithm identifying a subset of EV biomarkers that allowed the detection of multiple early-stage cancers including pancreatic, ovarian and bladder cancers^30^. In this study, we further refine our approach to specifically identify PDAC with early-stage cancers using EVs derived from blood plasma (ExoVita™ Pancreas assay). The developed classifier achieved a performance of 90% sensitivity and 92.8% specificity on an independent validation cohort (30 stage I and II PDAC cases, 83 controls) thus providing an advance in the fight against this deadly disease.

## Methods

### Specimen collection

The specimens for the training set were obtained from commercial biorepositories (ProteoGenex, Culver City, CA; DxBiosamples, San Diego, CA; Discovery Life Sciences, Huntsville, AL; Fox Chase Cancer Center, Philadelphia, PA; iSpecimen, Lexington MA; Tissue for Research, Newmarket, England). The specimens for the validation set were obtained from the Oregon Health and Sciences University, Portland, OR; Biotheme, Plantation, FL; Proteogenex, Culver city, CA; and iSpecimen, Lexington MA. Details on the specimens including phenotypical information are shown in Supplementary Data 1 and 2. Whole venous blood was collected and processed to plasma under appropriate Institutional Review Board/Independent Ethical Committee approval, and all patients filed informed consent. In particular, the specimens obtained from Oregon Health & Science University (OHSU) were collected through the Oregon Pancreas Tissue Registry (IRB00003609). Informed consent was obtained from all subjects and all experimental protocols were approved by the OHSU Institutional Review Board. All methods were carried out in accordance with relevant guidelines and regulations. All PDAC cases with a pathologically confirmed diagnosis of cancer were treatment-naïve (prior to surgery, local, and/or systemic anticancer therapy) at the time of blood collection. The information regarding cohort demographics, staging (per 7th edition AJCC), pathology and surgical status was provided by the biorepositories and reviewed for accuracy by a consulting physician. The training set was comprised of 105 PDAC cases and 545 controls while the validation set was composed of 30 PDAC confirmed cases and 83 controls matching the criteria for the training set while also including 11 patients with presentations of either chronic or acute pancreatitis. All patient samples were collected in K_2_EDTA plasma vacutainer tubes.

### Isolation of EVs using the Verita™ Platform

EVs, including exosomes, were extracted from plasma using an AC Electrokinetic (ACE)-based isolation platform (Biological Dynamics, CA, USA)^29,30^. The process starts with 240 µL of undiluted plasma from each patient and then it is introduced into a Verita™ chip, and an electrical signal of 7 Vpp and 14 KHz was applied while flowing the plasma across the chip at 3 µL/min for 120 min. EVs were captured onto the energized microelectrode array, and unbound materials were washed off the chip with Elution Buffer I (Biological Dynamics) for 30 min at 3 µL/min. The electrical signal was turned off, releasing EVs into the solution remaining on the chip (35 µL), which was then collected, and this solution containing purified, concentrated/eluted EVs was used directly for further analysis.

### EV-protein biomarker analysis

Verita-isolated EV samples were used directly in commercial multiplex immunoassays to quantify the presence of marker proteins. In brief, 25 µL of each purified EV sample was used for analysis by each of three different bead-based immunoassay kits, according to the manufacturer’s directions for each kit (Human Circulating Biomarker Magnetic Bead Panel 1 (Cat # HCCBP1MAG-58K), Human Angiogenesis Magnetic Bead Panel 2 (Cat # HANG2MAG-12K), and Human Circulating Cancer Biomarker Panel 3 (Cat # HCCBP3MAG-58K); Millipore Sigma, Burlington, MA). Protein biomarker concentration was assessed using the MAGPIX system (Luminex Corp, Austin, TX) according to the manufacturer’s protocols. Belysa software v. 3.0 (EMD Millipore) was used to determine final protein concentrations from the calibration curves. In cases with missing values or results below the lower limit of detection (LLoD), values were set (imputed) to the LLoD.

### ExoVita pancreas classifier development

The initial determination of the biomarker set, subsequent algorithm selection, biomarker feature selection, and perturbation analysis were determined from the Training set exclusively using cross-validation as described below. The final chosen model, limited to the subset of features selected, was then locked in the Training set, and applied to the independent Validation set.

#### Initial determination of biomarker set

The analysis dataset began with 52 EV-protein biomarkers under consideration for potential use as features. Prior to machine-learning (ML) analysis, two filter-based feature selection methods were used to determine a subset of potentially informative and reliably measurable biomarkers as the prospective set of candidate features: (1) Biomarkers used in the model were first reduced to a set of 36 proteins by restricting candidate biomarkers to those which could be reliably measured, as determined by ≤50% of patients having values imputed to the lower limit of detection (LLoD). (2) From the reduced set of 36 EV-protein markers, biomarker pairs with >0.9 Pearson-*r* correlation coefficients (see Supplementary Data 3) were subjected to Kolmogorov–Smirnov test for binary response indicating the presence or absence of cancer. The biomarker with the highest *P* value within the pair, corresponding to lower differentiation in respect to the binary case-control response, was removed from consideration (a total of 2 biomarkers were removed). The resulting 34 EV-protein biomarkers comprised the prospective set of candidate features for ML analysis.

#### Algorithm selection, biomarker feature selection, and training performance

The ExoVita Pancreas ML classifier development process used 100 repetitions of fivefold cross-validation stratified by the binary presence of cancer response to determine the optimal combination of feature transformation, algorithm, algorithm-specific hyper-parameters, and candidate biomarkers that maximized the partial AUC (pAUC). The pAUC is defined as the area under the ROC curve for specificities ranging from 90 to 100%. All combinations were applied within the folds of cross-validation, avoiding data leakage from the simulated training sets into their corresponding simulated validation set folds. Performance was evaluated within the simulated validation sets and subsequently aggregated as the average performance over all simulated validation sets. Further filter-based feature selection, identical to what was used to determine the initial set of candidate biomarkers, was applied within the cross-validation procedure to explore lower acceptable imputation values both up to the 50% level, and lower acceptable Pearson-*r* correlation coefficients up to the 0.9 value. The set of different algorithms considered included boosted tree-based methods, regularized logistic regression, neural networks, random forest, and support vector machines. Feature transformations under consideration were specific to the choice of algorithm. These included discretizing transformers, nonlinear functions, scaling transformations, and standardization techniques where appropriate^37^.

Feature (biomarker) importance was assessed concurrently with cross-validation performance using a model-agnostic permutation-based method. Within each simulated Training-Validation set pair, the model was fit in Training set and performance was assessed in the matched Validation set. Three measures of performance were considered as baselines: the partial AUC for specificity values ranging from 90 to 100%, the overall AUC, and the sensitivity of the model. Subsequently, for each feature used in the model, the values of said feature were randomly shuffled ten times across all observations in the Validation set and the average loss in performance from baseline for each of the three metrics across the ten random shuffles was computed as the feature permutation score. This process was repeated and averaged across each simulated Training-Validation set pair and all repetitions of cross-validation. Higher permutation score values indicated greater loss in performance and, therefore, greater importance to the model (Supplementary Data 4).

#### Algorithm robustness to perturbation

Following the methodology used to assess model robustness in ref. ^38^, prospective candidate models with high performance in the Training set cross-validation procedure were fit to the entirety of the Training set and subjected to perturbation analysis to determine their robustness to empirically observed within-patient variation at the biomarker level.

Average within-patient coefficients of variation (CVs) were calculated for each biomarker using all measurements used to build the analysis dataset. To perform this calculation, all measurements indicating no presence were set to the biomarker-specific LLoD and the within-patient CVs were calculated for each patient for each biomarker. These CVs were then averaged across all patients for each biomarker to obtain the mean within-patient CV for each biomarker (Supplementary Data 5).

Using these CV values, the measurements were then altered such that each observed data point was randomly varied up to +/− the input CV% in 68% of the sample data (equivalent to observing at 1 std dev in a bell curve) and between +/− CV% to 2CV% in the remaining 32% of the data (a more far-off scenario representing data between 1 and 2 std dev in a bell curve). This process was repeated 100 times and used to create 100 perturbed in silico test sets to assess the robustness of the candidate models determined to be of most interest to empirically observed within-patient biomarker variation.

#### Choice of the optimal algorithm

The final choice of algorithm aimed to balance performance in the training set cross-validation procedure, including robustness to biomarker perturbation, parsimony, and interpretability. This was determined to be a logistic regression model using recursive feature elimination^37^ to select a subset of seven, Log2 transformed EV-protein biomarkers subject to a ≤30% imputed value cutoff and a ≤0.5 Pearson-*r* correlation coefficient biomarker filter.

The locked ExoVita Pancreas classifier used is based on a logistic regression algorithm. The logistic regression model employs a logistic function, or sigmoid function, to map a linear combination of input features to a value between 0 and 1. In our case, we call this value the ExoVita Score and chose a cutoff point within the 0 to 1 range to achieve our desired specificity level of 91% in the Training set. The cutoff is then fixed from the training set and used in the independent Validation set to classify tested samples as high likelihood or low likelihood for PDAC.

### Reporting summary

Further information on research design is available in the Nature Portfolio Reporting Summary linked to this article.

## Results

### Biomarker and model selection from the training set

**Fig. 1:**
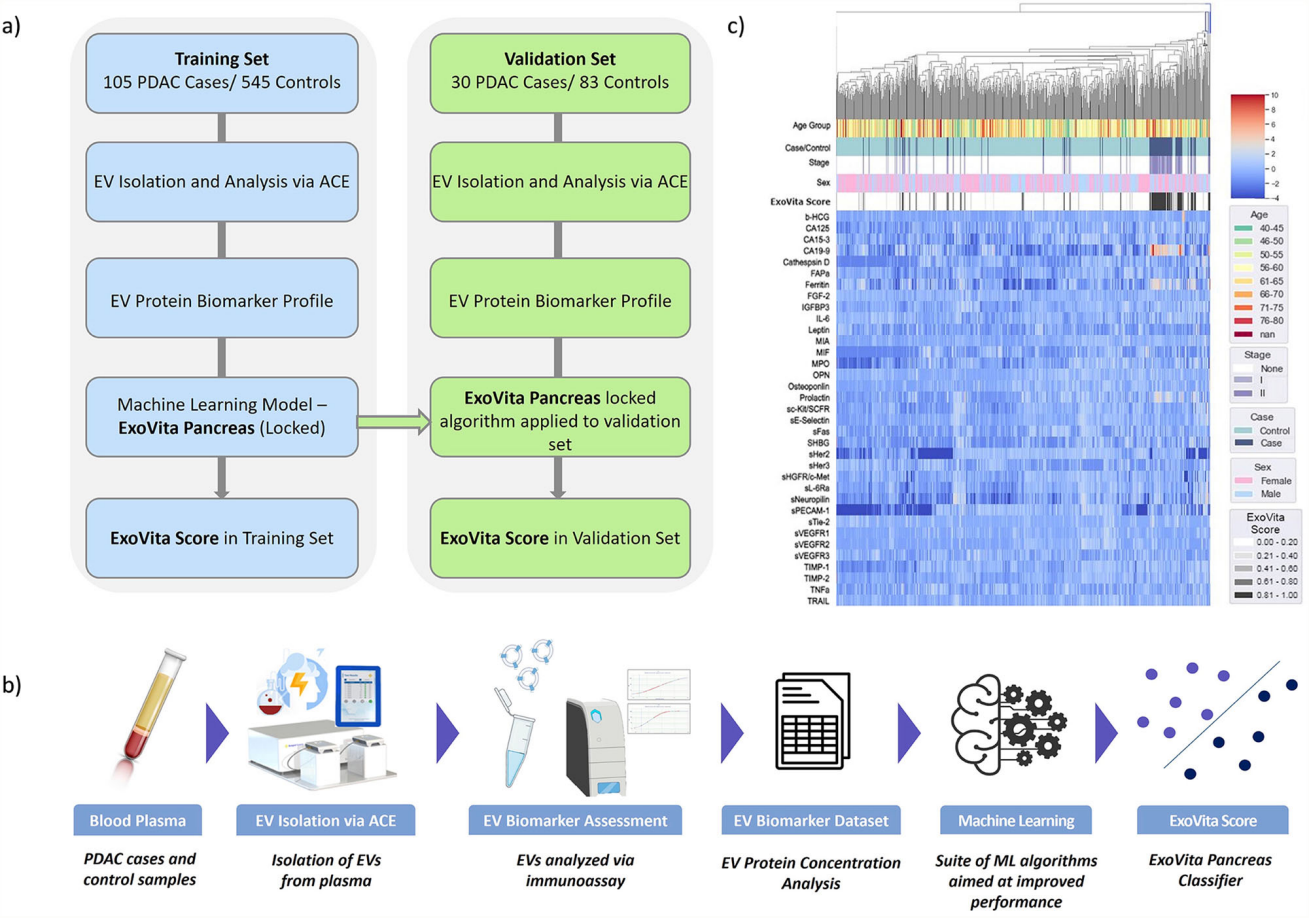
Schematic of the overall approach. **a** The training cohort included a total of 650 patients. After Extracellular Vesicle (EV) isolation and EV biomarker profile, machine learning (ML) was used to generate the ExoVita Pancreas classifier. The locked classifier was then applied to an independent validation set of 113 patients. **b** Data acquisition and analysis workflow starting with blood plasma followed by EV isolation and EV-protein measurement. The data was processed to determine the most suitable approach for the development of a Pancreatic Ductal Adenocarcinoma (PDAC) classifier. **c** Heatmap representation of the differences in EV-protein expression between the cases and control cohorts. The data was transformed by taking the case and control groups, dividing the mean of the control group across individual markers, and then applying a log2 conversion. This figure was created with assistance from biorender.com.

To evaluate EV-protein concentrations, ACE methodology was used to isolate EVs from blood plasma in a training cohort of 650 patients, including 105 pathologically confirmed, treatment-naive, stage I or II PDAC cases, and 545 control patients with no history of cancer for the training set (Table 1 and Fig. 1a). Following isolation, EVs were analyzed using multiplex immunoassays to determine the concentrations of individual proteins associated with the EVs (Fig. 1b, c). Through the development of the machine-learning (ML) classifier that provided optimal diagnostic performance for detection of early-stage PDAC, seven EV-protein biomarkers were selected (Supplementary Data 4 and 6).

**Table 1:** Patient demographics for training and validation sets.

Cohort	Training set (*N* = 650)				Validation set (*N* = 113)			
	*N*	Median age (range)	Female	Male	*N*	Median age (range)	Female	Male
PDAC cases	105	62 (38–82)	62	43	30	70 (45–85)	15	15
*Stage I*	39	64 (47–74)	28	11	10	68 (51–83)	6	4
*Stage II*	66	61 (38–82)	34	32	20	74 (45–85)	9	11
Controls	545	59 (40–84)	312	233	83	59 (45–82)	59	24

### Receiver operating characteristic curve and threshold determination in the training set

**Fig. 2:**
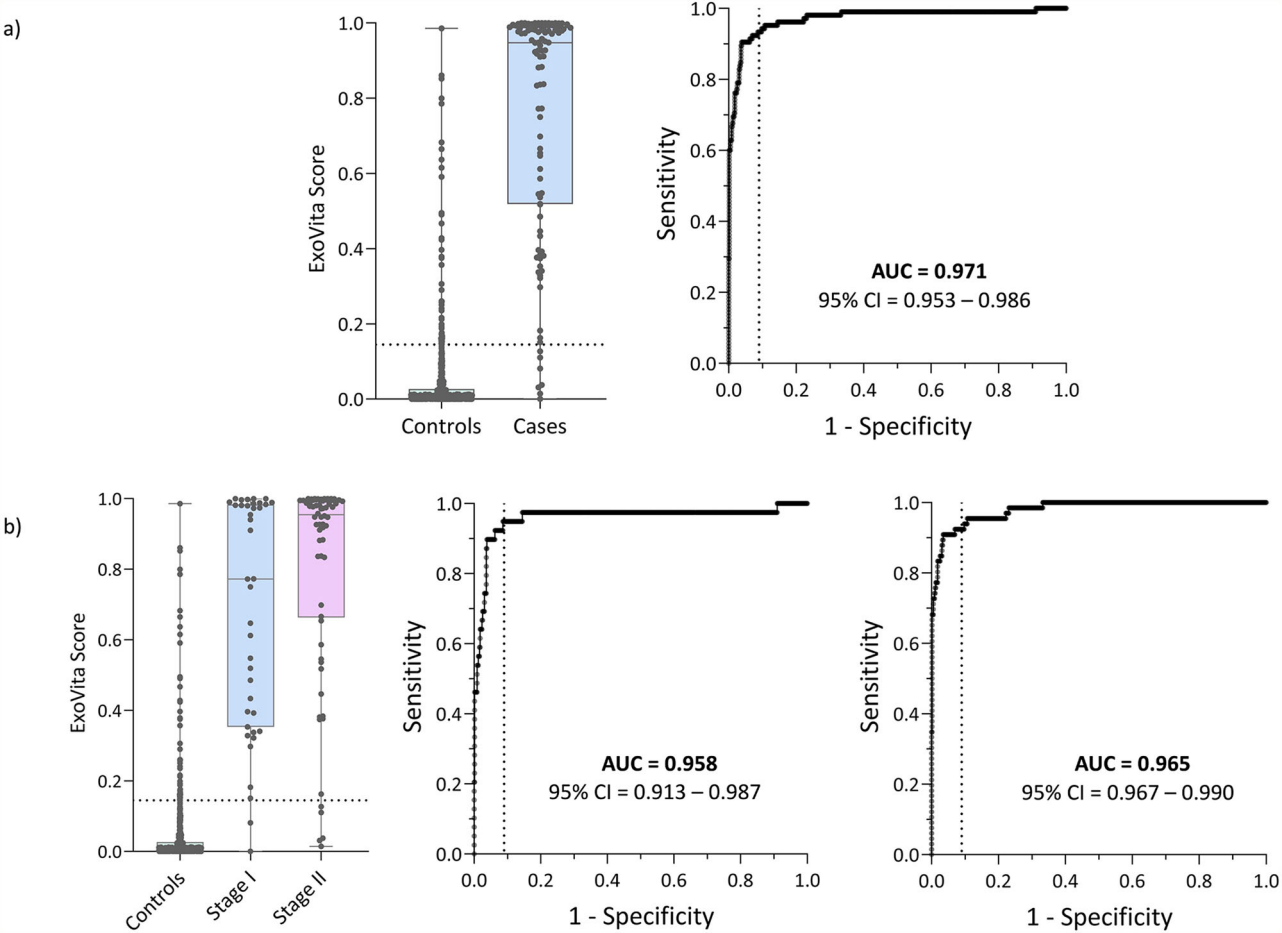
Performance of ExoVita pancreas in training set. **a** ExoVita Score distribution across the case (*n* = 105) and control (*n* = 545) cohorts in the training set. The box plots represent the median and interquartile range with each dot overlayed representing a patient in the training set. The whiskers represent the min and max of the distribution. **b** Further split of the ExoVita Score for the stage I and II cases in the training dataset. The box plots represent the median and interquartile range with each dot overlayed representing a patient in the training set. The whiskers represent the min and max of the distribution. The dotted line in both (**a**, **b**) represents the threshold selected to obtain 91% specificity as demonstrated on the receiver operating characteristic (ROC) curves displayed to the right of each panel. The 95% CI on the AUC is determined by bootstrapping in a total of 2000 repetitions.

The classifier outputs a numerical score (ExoVita Score) between 0 and 1, which was used to compare the case and control populations (Table 1), with scores analyzed to create a receiver operating characteristic (ROC) curve (Fig. 2a). The area under the ROC curve (AUC) of the ExoVita Pancreas classifier on the training set was 0.971 (95% CI: 0.953–0.986; Fig. 2a). The AUC obtained for the stage I cases vs the controls was 0.958 (95% CI: 0.913–0.987) and for the stage II cases vs the controls was 0.979 (95% CI: 0.967–0.990) as shown in Fig. 2b. Because the intended use population for the test is individuals with a high risk for PDAC, the threshold was set at a 91% specificity level yielding a sensitivity in the training set of 93.3% (95% CI: 86.9–96.7), with stage I sensitivity of 94.9% (95% CI: 83.1–98.6), and stage II sensitivity of 92.4% (95% CI: 83.5–96.7) as shown in Table 2. A detailed record of all possible ExoVita Score cutoffs (thresholds) from the training set and their respective performance in both the training and validation set are shown in Supplementary Data 7.

**Table 2:** Performance of ExoVita pancreas classifier in the training and validation sets.

Cohort	Training set (95% CI)^a^			Validation set (95% CI)^a^		
	*N*	Sensitivity, %	Specificity, %	*N*	Sensitivity, %	Specificity, %
PDAC cases	105	93.3 (86.9–96.7)		30	90.0 (74.4–96.5)	
*Stage I*	39	94.9 (83.1–98.6)		10	100.0 (72.2–100)	
*Stage II*	66	92.4 (83.5–96.7)		20	85.0 (64.0–94.8)	
Controls	545		91.0 (88.3–93.1)	83		92.8 (85.1–96.6)

^a^Two-sided 95% Wilson confidence intervals.

### Perturbation analysis

To evaluate the robustness of the ExoVita Pancreas classifier, perturbation analysis of the assay was conducted by intentionally altering the data based on experimental observations of variations in the EV-protein readings to see how those variations would affect the output of the model. To accomplish this, the reproducibility of each of the seven biomarkers was analyzed individually using the full cohort of patient data to identify the mean and median CVs (Supplementary Data 6). Details on the perturbation analysis are described in “Methods”.

Following the methodology used by Chalasani et al.^38^, a total of 100 perturbed test datasets were created to assess the robustness of the model against the algorithm developed in the training set (Fig. 2). The average AUC obtained across 100 perturbation sets was 0.967 with an average sensitivity of 92.7% and an average specificity of 89.4% (Table 3 and Supplementary Data 8).

**Table 3:** Performance outcomes from 100 simulated, in silico, datasets for perturbation analysis.

Performance metric	Mean value	Minimum value	Maximum value
AUC	0.967	0.962	0.971
Specificity	89.4%	87.7%	90.9%
Overall sensitivity	92.7%	89.6%	96.0%
*Stage I sensitivity*	92.6%	85.7%	95.9%
*Stage II sensitivity*	92.8%	89.5%	96.1%

### ExoVita Pancreas classifier evaluation using an independent validation cohort

**Fig. 3:**
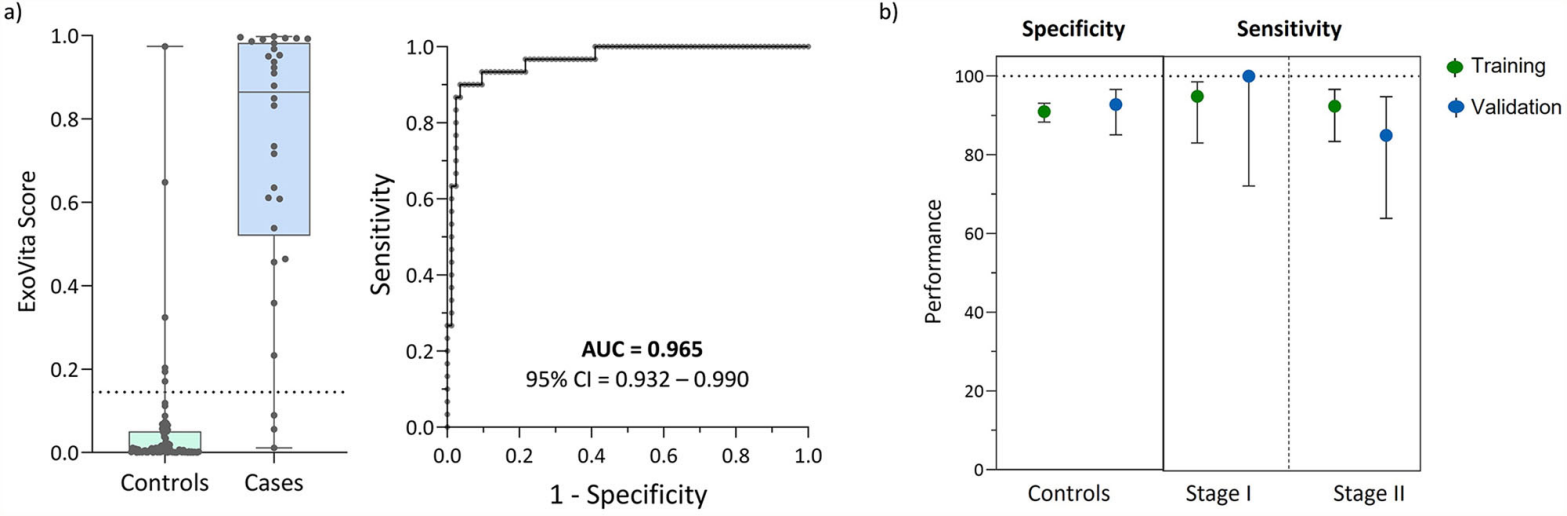
ExoVita pancreas performance in the independent validation set. **a** ExoVita Score distribution across the case (*n* = 30) and control (*n* = 83) cohorts in the validation set. The box plots represent the median and interquartile range with each dot overlayed representing a patient in the validation set. The whiskers represent the min and max of the distribution. The dotted line represents the threshold selected to obtain 91% specificity from the training set. The receiver operating characteristic (ROC) curve analysis for the validation set is shown on the right. The 95% CI on the Area Under the Curve (AUC) is determined by bootstrapping in a total of 2000 repetitions. **b** Comparison of performance for the training and validation cohorts broken down by stage using the ExoVita Score threshold selected in the training set. The error bars represent the two-sided 95% Wilson confidence interval.

Based on the performance of the training set, a second set of samples, comprising 30 stage I and II cases and 83 controls, were tested in a blinded fashion and served as an independent validation set for ExoVita Pancreas. The control dataset included 11 patients with presentation of pancreatitis (as shown in Supplementary Data 2). EVs were isolated from patient samples, and biomarker concentrations measured using the same methods as those used for the training cohort. Following data collection, the ExoVita Pancreas classifier algorithm, using the previously selected cutoff value based on the 91% specificity from the training set (as shown in Fig. 2), was applied to the validation set. After the ExoVita Scores were computed for each patient, the blind was removed, and the test calls were then compared to the known patient condition (case, control) to evaluate the performance of the classifier in the validation set (Table 2). We found that the test performance for this independent validation set was 90.0% sensitivity (95% CI: 74.4– 96.5) and 92.8% specificity (95% CI: 85.1–96.6), which was well within the expected outcomes from the training set and confirmed the potential utility of the ExoVita Pancreas classifier (Fig. 3).

## Discussion

Pancreatic cancer is a deadly disease with high mortality, despite having a relatively low prevalence when compared to other cancers. It is currently one of the leading causes of cancer-related deaths in the United States and both the incidence and death rates are expected to grow^2,39,40^. The poor prognosis for pancreatic cancer stems from the combination of inadequate diagnostic methods at the early stages and a lack of curative strategies for the late stages^5^. Stage I or earlier PDAC patients have 5-year survival rates as high as 80%^41^, while survival rates for stage IV patients plummet to about 3%^12,42^. While carbohydrate antigen 19-9 (CA19-9) is FDA-approved and widely used to aid in the diagnosis of pancreatic cancer^43^, it lacks the necessary performance features for surveillance^44–46^. A contribution by Poruk et al.^47^ showed the aggregated (from multiple studies) performance of CA19-9 as 78.2% sensitivity and 82.8% specificity. From the same publication, the aggregate performance of another commonly used marker for pancreatic cancer, carcinoembryonic antigen (CEA), is 44.2% sensitivity and 84.8% specificity. A more recent publication showed sensitivities for CA19-9 of 64.7% when comparing resectable pancreatic cancer cases to healthy controls and 46.5% to when compared to pancreatitis patients, all at a 99% specificity^48^. Therefore, there is a clinical need for a more effective PDAC detection test and more responsive biomarkers that can provide early disease detection.

Numerous liquid biopsy tests have been emerging that utilize cell-free DNA as an analyte^19,49–53^. However, for individuals not previously diagnosed with cancer, these diagnostic tests may yield high specificities, but fail to demonstrate adequate sensitivities for early-stage disease. Indeed, many of these tests show sensitivity sufficient to detect only late-stage disease (stage III/IV), when tumor burden has increased, symptoms have intensified, and curative options have narrowed. One recent proteomics-based approach has been shown with the possibility of detecting PDAC in all stages; however, interpretation of its efficacy is hindered by the removal of indeterminate patients from the analysis and from dependency on detection of CA19-9 expression using conventional methodologies^54^. In high-risk settings, and therefore likely enhanced prevalence cohorts, higher sensitivity surveillance is likely to produce improved early detection outcomes.

A recent study by Nakamura et al. has shown how exosome transcriptomic signatures can be used in early pancreatic cancer detection^55^ while an earlier pilot study using the approach described here showed that a set of 13 EV-protein biomarkers could detect the presence of early-stage pancreatic, ovarian and bladder cancers in comparison to healthy controls in the context of a multi-cancer early detection (MCED)-type test^30^. For this study, the cohort size was greatly expanded to enable discrimination of early-stage PDAC, focused to the context of a single-cancer test, and includes both training and blinded independent validation cohorts. The larger training cohort permitted refinement of the algorithm while the validation set afforded for an independent evaluation of the developed classifier.

This work demonstrates that EV-protein biomarkers can be used to detect PDAC at its earliest stages while optimizing for high sensitivity. One advantage for use of EV biomarkers in liquid biopsy tests is that EVs are generated during all stages of cell transformation, from normal to tumor cell, and their release into circulation is often accelerated during the process of tumorgenesis^25,26,56^. Nascent tumors in situ may release EVs into circulation long before CTCs and ctDNA can be measured in blood, and EV presence is not dependent on cell death or necrosis^57^. In addition, EVs regulate cancer progression as well as transfer oncogenic proteins and nucleic acids during their interactions with the tumor microenvironment, suggesting that these markers are not simply related to inflammatory responses^58^.

EVs have been shown to play active roles in communication between tumor and stroma, expressing integrins that promote metastasis; they also play roles in immune response inhibition in cancer^59,60^ It is thus likely that EVs from both tumor and stroma will carry protein markers whose levels are altered during tumorigenesis. Proteins with roles in angiogenesis (such as ferritin, CA15-5, and leptin) are likely to be carried by EVs from tumor cells to endothelial cells. Other proteins, such as CA19-9 with roles in matrix remodeling, are also likely to be on EVs targeting the stroma. As in our earlier study^30^, markers relating to all aspects of tumor progression and function are represented, but with less overlap. CA19-9, ferritin, and HER3 can function as cancer drivers, and CA15-3, ferritin, leptin, and other markers used here are implicated in pathways affecting apoptosis, angiogenesis, and metastasis. Leptin, ferritin, FGF2, and prolactin may potentiate cancer stem cell formation; ferritin also impacts pathways leading to the EMT transition. Both ferritin and leptin are linked to the immune response. In this light, choice of a specific marker may not be as crucial to the test as is sufficient diversity of marker roles to cover the full range of tumor phenotypes, phases, and growth patterns^61–66^.

Currently, there are no screening strategies recommended to identify PDAC in the general population and strategies are limited for high-risk populations. These include the CAPS Consortium recommendation for annual screening with endoscopic ultrasonography (EUS) and/or MRI/magnetic resonance cholangiopancreatography for those individuals at high risk for PDAC^2,10,18,67^. A blood-based test may be more accessible for populations at high risk for PDAC, since patients could potentially use blood draw centers close to home or work, as well as mobile phlebotomy clinics. This could have broad implications for patients living in medically underserved areas or with economic impediments to healthcare access^68^.

With an aim to develop a more accessible, blood-based screening test for populations at high risk for PDAC, it was investigated here if a test integrating information from EV-protein biomarkers might have sufficient sensitivity to detect the cancer at its earliest stages. Using EVs purified from patient plasma, biomarker proteins were collectively quantified, and machine learning utilized to assign a total score (ExoVita Score) to each patient. By selecting an ExoVita Score threshold that achieves high sensitivity it will be possible to detect an increased number of cancer cases and bring them into the continuum of care earlier. In this study, the ExoVita Pancreas classifier demonstrated differentiation between early-stage PDAC (stages I/II) and healthy control patients using a model comprised of 7 EV-protein biomarkers yielding an AUC of 0.971 with a sensitivity of 93.3% at a specificity of 91.0%. Furthermore, the performance of the classifier is confirmed using an independent validation set showing both >90% sensitivity and specificity as shown in Table 2. Importantly, to better reflect future testing circumstances, a proportion of the control patients present certain conditions, as shown in the medical history and noted condition columns on Supplementary Data 1 and 2. The training set included 62 patients with either type-2 diabetes mellitus (T2DM) or pancreatitis while the validation set included 15 control patients with either T2DM or either acute or chronic pancreatitis. In the validation set, the classifier yielded just one control patient as positive from the noted conditions cohort. Because pancreatitis is a high-risk condition for PDAC, it is possible that this individual had very early, as-yet undiagnosed PDAC malignancy. Unfortunately, as direct access to the patient was not available, this could not be further investigated^69^. Overall, the results suggest that the ExoVita Pancreas test may be useful in discriminating the presence of very early PDAC.

For the first implementation of this ExoVita Pancreas test, the intended population is patients with higher-than-average risk for PDAC, including those with familial risk due to germline mutations, a history of pancreatitis, those with intraductal papillary mucinous neoplasms (IPMNs), and patients over 50 years of age with new-onset diabetes (NOD)^9^. The criteria for defining high-risk populations remain controversial as a general consensus from different guidelines has been accepted, but is not well-documented. For example, only recently have sequencing tests become more accessible to test patients with germline mutations at risk for PDAC. Given the increase in testing, long-term data will be required to capture the accuracy of these relative risks within these populations. In addition, a blood-based test could help bridge the gap of healthcare disparities. By offering high-risk patients a high-sensitivity test with easier access, more routine assessments can be made that aim to mitigate the mortality of PDAC while avoiding the enormous healthcare costs associated with false test results when screening an average-risk population. It is expected that these patients should receive standard-of-care testing in parallel with a blood-based test that can enhance overall clinical utility during patient care.

There are multiple limitations of this study. First, the case cohort is a relatively homogenous population that does not reflect diverse groups in the real world (see Supplementary Data 1 and 2). Second, this is a retrospective case-control study with no longitudinal follow-up data to provide information about treatment outcomes or the future appearance of pancreatic disease in the control patients. Third, cases with presentations of advanced PDAC (stages III and IV) were purposedly excluded, whereas in real life it is expected that advanced cases will be discovered.

In summary, this contribution describes an effective, high-sensitivity test to identify early-stage PDAC using an EV/exosome-isolating liquid biopsy platform. ACE technology was utilized to isolate EVs from patient plasma followed by analysis of EV-protein levels for a panel of seven biomarkers, and machine learning was employed to establish an algorithm identifying PDAC with high sensitivity and specificity. This approach is scalable and cost-effective and may ultimately lead to a more accessible screening application for PDAC. Further investigations on this topic are underway including a prospective, multi-center, observational registry study—ExoLuminate Study—to evaluate patients at high risk for PDAC (NCT0562552).

### Supplementary information

Supplementary Data 1

Supplementary Data 2

Supplementary Data 3

Supplementary Data 4

Supplementary Data 5

Supplementary Data 6

Supplementary Data 7

Supplementary Data 8

Description of Additional Supplementary Files

Reporting Summary

## Data Availability

Data supporting the findings of the study referenced in this paper are included herein and in the Supplementary Data. Source data for Figs. 1–3 can be found in Supplementary Data 1, 2, and 7 files. Requests to access additional datasets beyond that available herein will require consideration by the authors (including with respect to intended use, and intellectual property and confidentiality requirements). Requests for such data should be directed to the corresponding authors.

## Code availability

The code used in this study is proprietary information and may be used to pursue regulatory approvals and to allow for proper IP protection. Interested researchers can contact the corresponding authors for questions on its status and access. A code or data use agreement may be required to protect intellectual property.
