# Supplementary material for: Development of a blood-based extracellular vesicle classifier for detection of early-stage pancreatic ductal adenocarcinoma
Source: Commun Med (Lond). 2023 Oct 19;3:146. doi: 10.1038/s43856-023-00351-4 (PMC10587093; doi:10.1038/s43856-023-00351-4)
Supplement: Supplementary file 9 — Description of Additional Supplementary Files [file 43856_2023_351_MOESM9_ESM.pdf]

Description of Additional Supplementary Files

File name: Supplementary Dataset 1

Description: Demographic and EV Protein Readings for the Training Cohort.

File name: Supplementary Dataset 2

Description: Demographic and EV Protein Readings for the Validation Cohort.

File name: Supplementary Dataset 3

Description: Pearson-r Correlation Coefficients between EV protein markers.

File name: Supplementary Dataset 4

Description: Permutation Feature Importance in Cross-Validation for the Training Set in ExoVita Pancreas Classifier.

File name: Supplementary Dataset 5

Description: Mean and Median CV Values in EV Protein Biomarkers for Perturbation Analysis.

File name: Supplementary Dataset 6

Description: ExoVita Pancreas Biomarker Information and Relevance for Cancer.

File name: Supplementary Dataset 7

Description: Performance of ExoVita O=Pancreas Classifier at All Possible Thresholds (Cutoffs) from the Training Set.

File name: Supplementary Dataset 8

Description: Perturbation Analysis Probability and 95% Confidence Interval for Each Subject in Each Perturbation Set.
