# Supplementary material for: Development of a blood-based extracellular vesicle classifier for detection of early-stage pancreatic ductal adenocarcinoma
Source: Commun Med (Lond). 2023 Oct 19;3:146. doi: 10.1038/s43856-023-00351-4 (PMC10587093; doi:10.1038/s43856-023-00351-4)
Supplement: Supplementary file 10 — Reporting Summary [file 43856_2023_351_MOESM10_ESM.pdf]

nature portfolio

Corresponding author(s): Paul R. Billings

Last updated by author(s): Jul 29, 2023

Reporting Summary

Nature Portfolio wishes to improve the reproducibility of the work that we publish. This form provides structure for consistency and transparency in reporting. For further information on Nature Portfolio policies, see our Editorial Policies and the Editorial Policy Checklist.

Statistics

For all statistical analyses, confirm that the following items are present in the figure legend, table legend, main text, or Methods section.

n/a	Confirmed	
☐	☒	The exact sample size (*n*) for each experimental group/condition, given as a discrete number and unit of measurement
☐	☒	A statement on whether measurements were taken from distinct samples or whether the same sample was measured repeatedly
☐	☒	The statistical test(s) used AND whether they are one- or two-sided<br>*Only common tests should be described solely by name; describe more complex techniques in the Methods section.*
☐	☒	A description of all covariates tested
☐	☒	A description of any assumptions or corrections, such as tests of normality and adjustment for multiple comparisons
☒	☐	A full description of the statistical parameters including central tendency (e.g. means) or other basic estimates (e.g. regression coefficient) AND variation (e.g. standard deviation) or associated estimates of uncertainty (e.g. confidence intervals)
☐	☒	For null hypothesis testing, the test statistic (e.g. F, t, r) with confidence intervals, effect sizes, degrees of freedom and P value noted<br>*Give P values as exact values whenever suitable.*
☒	☐	For Bayesian analysis, information on the choice of priors and Markov chain Monte Carlo settings
☐	☒	For hierarchical and complex designs, identification of the appropriate level for tests and full reporting of outcomes
☐	☒	Estimates of effect sizes (e.g. Cohen's d, Pearson's r), indicating how they were calculated

Our web collection on statistics for biologists contains articles on many of the points above.

Software and code

Policy information about availability of computer code

Data collection	The EV proteomic data was collected in a commercial tool from Luminex (Xmap) and the quantification using an EMD millipore software (Belysa)
Data analysis	The analysis of the data was performed using a custom made code on the Microsoft Azure Machine Learning platform.

For manuscripts utilizing custom algorithms or software that are central to the research but not yet described in published literature, software must be made available to editors and reviewers. We strongly encourage code deposition in a community repository (e.g. GitHub). See the Nature Portfolio guidelines for submitting code & software for further information.

Data

Policy information about availability of data

All manuscripts must include a data availability statement. This statement should provide the following information, where applicable:

- Accession codes, unique identifiers, or web links for publicly available datasets
- A description of any restrictions on data availability
- For clinical datasets or third party data, please ensure that the statement adheres to our policy

All data supporting the findings of the study are available within the paper and its Supplementary Data. Specific data for figures 2 and 3 can be found in Supplementary Data 1 and 2 files. Requests to access additional datasets beyond those described in the manuscript, pertaining to the development of the Verita™ platform and ExoVita Pancreas test, will promptly undergo an internal review to verify whether the request is subject to any intellectual property or confidentiality

limitations. All additional released data and materials will be provided subject to a data transfer agreement. Requests should be directed to the corresponding author.

Human research participants

Policy information about studies involving human research participants and Sex and Gender in Research.

Reporting on sex and gender	Only sex information is collected from the participants
Population characteristics	Information about the sex and age distribution of the study populations is shown on the tables on the main manuscript. Subject by subject information is shown in the supplementary data including its source biorepository.
Recruitment	The samples were obtained from biorepositories based on the criteria outlined in the materials and methods section.
Ethics oversight	Samples were obtained from biorepositories which managed their internal IRBs

Note that full information on the approval of the study protocol must also be provided in the manuscript.

Field-specific reporting

Please select the one below that is the best fit for your research. If you are not sure, read the appropriate sections before making your selection.

☒ Life sciences ☐ Behavioural & social sciences ☐ Ecological, evolutionary & environmental sciences

For a reference copy of the document with all sections, see nature.com/documents/nr-reporting-summary-flat.pdf

Life sciences study design

All studies must disclose on these points even when the disclosure is negative.

Sample size	Sample size was not predetermined and depended upon subject availability.
Data exclusions	No subject was excluded from the study.
Replication	The EV proteomic analysis was run in triplicate, duplicate or singlicate depending on plasma volume availability.
Randomization	Samples were randomized for running in multiple days, batches, operators without distinction between cases and controls.
Blinding	Because this is case-control study, the investigators were not blinded to the true condition of each subject.

Reporting for specific materials, systems and methods

We require information from authors about some types of materials, experimental systems and methods used in many studies. Here, indicate whether each material, system or method listed is relevant to your study. If you are not sure if a list item applies to your research, read the appropriate section before selecting a response.

Materials & experimental systems

n/a	Involved in the study	
☒	☐	Antibodies
☒	☐	Eukaryotic cell lines
☒	☐	Palaeontology and archaeology
☒	☐	Animals and other organisms
☒	☐	Clinical data
☒	☐	Dual use research of concern

Methods

n/a	Involved in the study	
☒	☐	ChIP-seq
☒	☐	Flow cytometry
☒	☐	MRI-based neuroimaging
